# Patients with chronic hepatitis B who have persistently normal alanine aminotransferase or aged < 30 years may exhibit significant histologic damage

**DOI:** 10.1186/s12876-024-03208-9

**Published:** 2024-03-27

**Authors:** Sufang Wei, Qiuli Xie, Guichan Liao, Hongjie Chen, Meixin Hu, Xiaoli Lin, Hong Li, Jie Peng

**Affiliations:** 1 Department of Infectious Diseases, Shunde Hospital, Southern Medical University, Foshan, 528308 China; 2 Department of Infectious Diseases, State Key Laboratory of Organ Failure Research, Guangdong Provincial Key Laboratory of Viral Hepatitis Research, Nanfang Hospital, Southern Medical University, Guangzhou, 510080 China; 3 Department of Infectious Diseases, Nanfang Hospital, Southern Medical University, Guangzhou, 510080 China

**Keywords:** Hepatitis B, Chronic, Alanine transaminase, Age, Liver histology

## Abstract

**Background:**

The timing of antiviral therapy for chronic hepatitis B (CHB) patients with normal alanine transaminase (ALT) or aged < 30 years is still undetermined. We aimed to elucidate the correlation between liver histology, age, and ALT level in CHB patients and analyze the histological characteristics of the liver among patients with persistently normal ALT or aged < 30 years.

**Methods:**

A retrospective analysis was conducted on 697 treatment-naive CHB patients. Liver biopsies were performed, and significant histological damage was defined as the grade of liver inflammation ≥ G2 and/or fibrosis ≥ S2 based on the Scheuer scoring system.

**Results:**

The liver inflammation grades and fibrosis stages correlated positively with age, ALT, AST, GGT levels and negatively with the counts of PLT (all *p* < 0.050) in HBeAg-positive patients. Higher ALT levels and lower PLT counts were independently associated with significant liver inflammation and fibrosis in both HBeAg-positive and HBeAg-negative patients. Furthermore, among those with persistently normal ALT levels, the incidence of significant liver inflammation and fibrosis were 66.1% and 53.7% in HBeAg-positive groups, and 63.0% and 55.5% in HBeAg-negative groups. Moreover, there was no significant difference in the prevalence of significant liver damage between patients aged < 30 years and those aged ≥ 30 years, in both HBeAg-positive (≥ G2 or ≥ S2: 63.8% vs. 75.8%, *p* = 0.276) and HBeAg-negative (≥ G2 or ≥ S2: 65.9% vs. 72.5%, *p* = 0.504) groups, among patients with persistently normal ALT levels.

**Conclusions:**

A considerable proportion of CHB patients with persistently normal ALT, including those below the age of 30 years, exhibited significant histological damage. This highlights the importance of initiating early antiviral therapy for HBV-infected individuals, even in the absence of elevated ALT levels.

**Supplementary Information:**

The online version contains supplementary material available at 10.1186/s12876-024-03208-9.

## Introduction

Chronic hepatitis B virus (HBV) infection, which can lead to cirrhosis and hepatocellular carcinoma (HCC), remains a major public health concern [1]. Elimination of hepatogenic HBV or clearance of hepatitis B surface antigen (HBsAg), stalling disease progression, and preventing end-stage liver disease are the optimal goal, thus timely and effective antiviral therapy is crucial [2]. The WHO estimated that the number of people with chronic HBV infection was 296 million in 2019, with 1.5 million new infections per year [3]. And, there are approximately 86 million cases of chronic HBV infection in China, but only a meager 20 million individuals are receiving treatment, indicating a remarkably low rate of treatment [4]. Thus, achieving the 2016 WHO objective of eliminating chronic hepatitis B (CHB) by 2030 continues to pose a challenge in China [5].

Patients with chronic HBV infection who have normal alanine transaminase (ALT) levels were previously deemed to be in “immune-tolerant” or “inactive carrier” phase. Moreover, these individuals are typically not prescribed antiviral therapy, because their ALT levels within the normal range and hepatic damage is considered minimal. However, certain patients fall into an indeterminate phase because their HBV DNA and ALT levels do not align the conventional period [6, 7], leading to imprecise timing of treatment. Furthermore, accumulating evidence suggest that significant liver inflammation or fibrosis is not uncommon in patients with CHB who exhibit normal ALT levels [8, 9], and untreated CHB patients may be at a high risk of developing cirrhosis and HCC [10, 11]. As a result, the indications for antiviral treatment in CHB have been gradually expanded by national and international guidelines. However, the optimal timing of initiating antiviral therapy in CHB patients with normal ALT levels remains an unmet need.

Elder age has been established as a risk factor for the progression of liver disease [12]. According to AASLD 2018 guidelines, age over 40 years, particularly when companied by positive HBV DNA and family history of cirrhosis or HCC, is a major indicator for initiating antiviral treatment in CHB [13]. Studies have demonstrated that patients with CHB who are older than 30 years are more likely to experience histological damage and, therefore, are recommended for antiviral treatment [14–16]. The histological characteristics of CHB patients under the age of 30 have not been extensively studied. There has been an argument whether patients younger than 30 years with normal ALT level should be administrated antiviral treatment.

This retrospective study sought to explored the correlation between liver histology and age or ALT levels in CHB patients. In particularly, this study investigated the occurrence of significant histological damage in patients with persistently normal ALT or aged < 30 years.

## Materials and methods

### Patients

This retrospective study was conducted on patients with CHB who were admitted to Shunde Hospital of Southern Medical University from August 1999 to August 2003. Liver biopsy and pathological examination were performed on patients who did not receive antiviral treatment. “Persistently normal ALT” was defined as an ALT level < 40 U/L on at least 3 occasions over a 1- year period before liver biopsy. The inclusion criteria included: (1) Positive HBsAg for more than 6 months; (2) Positive HBV DNA; (3) Voluntarily undergoing liver biopsy; (4) Untreated patients. Patients were excluded if they met the following conditions: (1) Patients exposed to other viruses such as hepatitis C virus, hepatitis D virus, and human immunodeficiency virus; (2) Evidence of decompensated hepatic sclerosis, hepatocellular carcinoma, schistosomiasis liver disease, hemochromatosis and Wilson disease; (3) Patients had liver damage induced by other causes (cholestatic hepatitis, autoimmune hepatitis, alcoholic liver disease, and nonalcoholic fatty liver disease, etc.); (4) Undergoing liver transplantation prior to enrollment; (5) Coexistence of severe cardiovascular disease, kidney disease, and lung disease. As shown in Supplementary Fig. 1, finally, 697 patients were included in this study.

This study was approved by the Clinical Ethics Committee of Shunde Hospital of Southern Medical University (KYLS20230801). Written informed consent was obtained from all patients who underwent liver biopsy.

### Clinical parameters

Demographic data, including age and gender, were retrospectively collected from medical records. All patients underwent laboratory evaluation before liver biopsy. Biochemical parameters, including ALT, aspartate aminotransferase (AST), and gamma-glutamyl transferase (GGT), were determined. The upper limits of normal (ULN) of ALT was 40 IU/L for both males and females. Hepatitis B markers, including HBsAg, hepatitis B e-antigen (HBeAg), anti-HBe, and HBV DNA, were measured. HBV DNA was quantified by molecular hybridization, using the pre-2002 Digene Hybrid Capture II (HCII) test and the post-2002 polymerase chain reaction Cobas Monitor, and the lowest limitation of detection was 10,0000 copies/ml for Digene HCII and 10,000 copies/ml for Cobas Monitor. White blood cell count and platelet (PLT) counts were also recorded.

### Liver biopsy and histology

Liver biopsy whose major side effects were bleeding, pain, or pneumothorax, was performed under the guidance of ultrasound, and the specimens were evaluated by two experienced pathologists who were blinded to patients' identities. The Scheuer classification was adopted to assess liver inflammation and fibrosis. Liver inflammation grade ranged from G0 to G4 and fibrosis stage ranged from S0 to S4 [17]. G0 and G1 were considered no or mild liver inflammation, and ≥ G2 was considered significant liver inflammation. Similarly, no or mild liver fibrosis was defined as S0-1 and significant liver fibrosis was defined as ≥ S2, respectively.

### Statistical analysis

All statistical analyses were conducted using SPSS version 25.0 (SPSS Inc., Chicago, Illinois, USA). Data are expressed as mean ± SD or median (interquartile range) for continuous variables and number (percentage) for categorical variables. The chi-square test was used for comparing categorical variables. Mann–Whitney U or Kruskal–Wallis tests were used to compare non-normal variables between groups as appropriate. Correlation between serum parameters and liver histological grade was assessed using Spearman's rank correlation coefficient. Multivariate (binary) logistic regression analysis was performed in a forward method to identify factors independently associated with significant inflammation or fibrosis. All tests were performed two-tailed, and statistical significance was defined as *p* < 0.05.

## Results

### Clinical characteristics and liver histology of all patients

The clinical characteristics of all patients are summarized in Table 1. Among the 697 patients enrolled, 464 were HBeAg-positive and 233 were HBeAg-negative. HBV DNA quantification was performed in 198 HBeAg-positive and 58 HBeAg-negative patients (102 samples using Digene HCII test and 154 samples using Cobas Monitor test). Patients with HBeAg-negative had older age (31 vs. 27 years, *p* < 0.001) and a lower level of HBV DNA (6.1 vs. 7.3 log_10_ copies/mL, *p* < 0.001), than those with HBeAg-positive. Nevertheless, there were no substantial differences in ALT levels, GGT levels, and platelet counts between the two groups.

**Table 1:** Baseline characteristics and histologic outcomes of total patients

**Characteristics**	**HBeAg-positive** **(*n* = 464, 66.6%)**	**HBeAg-negative** **(*n* = 233, 33.4%)**	***p*-value**
Age, mean ± SD, years	27 ± 8	31 ± 9	< 0.001
Male, n, (%)	383(82.5)	203(87.1)	0.119
ALT, median (IQR), U/L	106 (40–300)	68 (28–231)	0.289
ALT ≤ ULN, n, %	117 (25.2)	92 (39.5)	
ALT 1–2 × ULN, n, %	72 (15.5)	30 (12.9)	
ALT ≥ 2 × ULN, n, %	275 (59.3)	111 (47.6)	
AST, median (IQR), U/L	74 (38–172)	56 (32–137)	0.940
GGT, median (IQR), U/L	52 (25–109)	58 (32–124)	0.054
WBC, median(IQR), × 10^9^/L	6.1 (4.9–7.1)	5.9 (5.1–6.9)	0.377
PLT, median (IQR), × 10^9^/L	170 (137–205)	158 (128–197)	0.105
HBV DNA, median (IQR), (log_10_ copies/mL)^a^	7.3 (6.1–8.3)	6.1 (4.9–7.0)	< 0.001
Inflammation n (%)			0.096
G0-1	78 (16.8)	54 (23.1)	
G2	192 (41.4)	92 (39.5)	
G3	135 (29.1)	60 (25.8)	
G4	59 (12.7)	27 (11.6)	
Fibrosis n (%)			0.395
S0-1	126 (27.2)	70 (30.0)	
S2	190 (40.9)	79 (33.9)	
S3	86 (18.5)	51 (21.9)	
S4	62 (13.4)	33 (14.2)	

The ULN for both ALT, AST was 40U/L, and the ULN for GGT was 60U/L

*IQR* Interquartile range, *ULN* Upper limit of normal, *ALT* Alanine transaminase, *AST* Aspirate aminotransferase, *GGT* Gamma-glutamyl transferase, *WBC* White blood cells, *PLT* Platelet, *HBV* hepatitis B virus

^a^The quantified detection of HBV DNA was available in a subset of 256 patients, of 198 patients were HBeAg-positive and 58 patients were HBeAg-negative, respectively

According to the ALT levels, patients were divided into three groups: group 1: ALT < 1 × ULN, group 2: ALT 1–2 × ULN, and group 3: ALT ≥ 2 × ULN. In HBeAg-positive group, 117 (25.2%), 72 (15.5%) and 275 (59.3%) were classified into ALT < 1 × ULN, ALT 1–2 × ULN and ALT ≥ 2 × ULN subgroups, respectively; while 92 (39.5%), 30 (12.9%) and 111 (47.6%) were categorized into ALT < 1 × ULN, ALT 1–2 × ULN and ALT ≥ 2 × ULN subgroups, respectively, in the HBeAg-negative group.

In the HBeAg-positive group, the distribution of liver inflammation grades was as follows: G0-1 = 78 (16.8%), G2 = 192 (41.4%), G3 = 135 (29.1%), and G4 = 59 (12.7%). Similarly, in the HBeAg-negative group, the distribution of liver inflammation grades was as follows: G0-1 = 54 (23.1%), G2 = 92 (39.5%), G3 = 60 (25.8%), and G4 = 27 (11.6%). No significant difference in the distribution of inflammation grades between the HBeAg-positive and negative groups (*p* = 0.096) was observed**.** The distribution of liver fibrosis stages was as follows: S0-1 = 126 (27.2%), S2 = 190 (40.9%), S3 = 86 (18.5%), and S4 = 62 (13.4%) in the HBeAg-positive group; S0-1 = 70 (30.0%), S2 = 79 (33.9%), S3 = 51 (21.9%), and S4 = 33 (14.2%) in the HBeAg-negative group. Similarly, the distribution of fibrosis stages was not markedly different between the HBeAg-positive and negative groups (*p* = 0.395).

According to the EASL 2017 guidelines [18], we further divided the 256 patients with quantitative HBV DNA data into four phases. The numbers of patients with chronic infection and chronic hepatitis were 16 and 78 in the HBeAg-positive group and 2 and 37 in the HBeAg-negative group, respectively. The distribution of liver inflammation grades and fibrosis stages is described in Supplementary Table 1.

### Correlation between the clinical parameters and the grades of liver inflammation and fibrosis

**Fig. 1:**
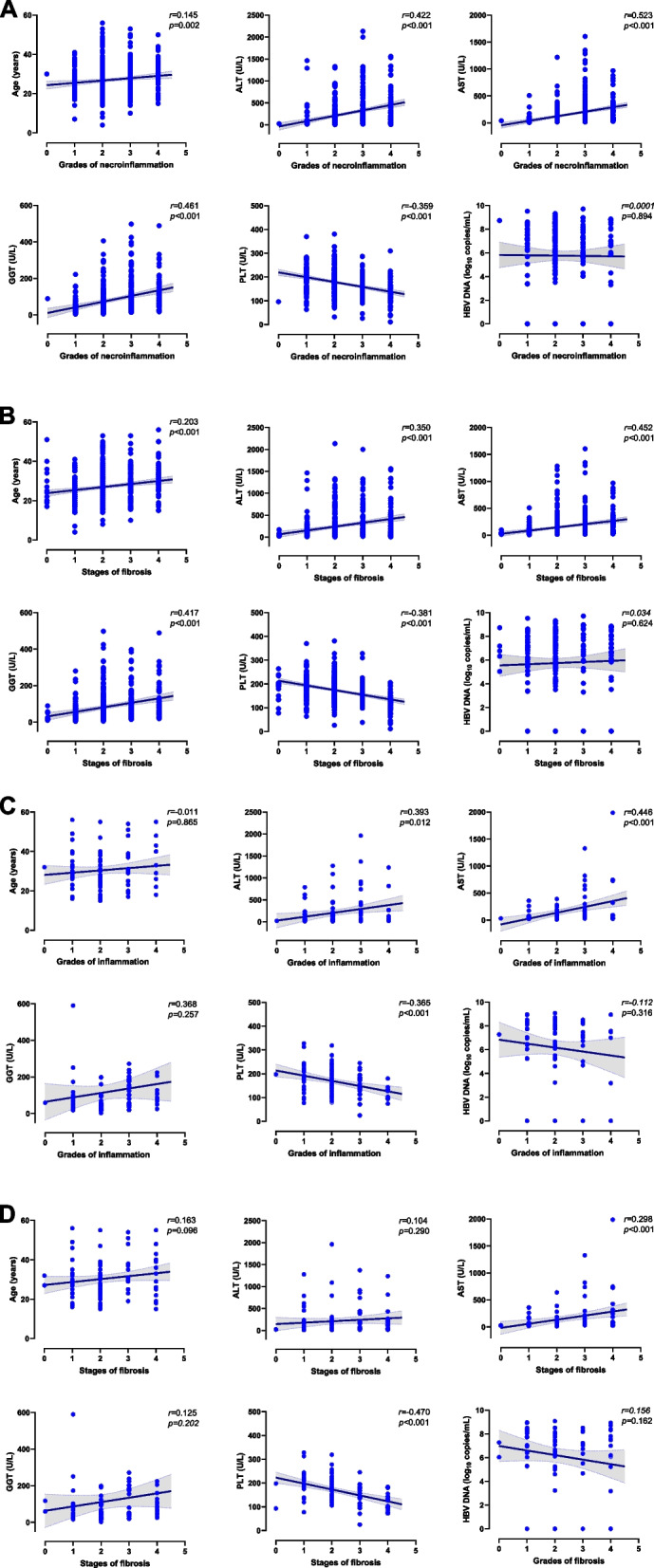
Correlation between age, serum markers and liver histology. **A** Correlation between age, serum markers, and inflammation grade in patients with HBeAg-positive CHB; **B** Correlation between age, serum markers, and fibrosis stage in patients with HBeAg-positive CHB; **C** Correlation between age, serum markers, and inflammation grade in patients with HBeAg-negative CHB; **D** Correlation between age, serum markers, and fibrosis stage in patients with HBeAg-negative CHB. The grey areas represent 95%CI confidence interval. ALT, alanine transaminase; AST, aspirate aminotransferase; GGT, gamma-glutamyl transferase; PLT, platelet

In the HBeAg-positive group, positive correlations were found between the grades of liver inflammation and age (*r* = 0.145, *p* = 0.002), ALT (*r* = 0.422, *p* < 0.001), AST (*r* = 0.523, *p* < 0.001), and GGT (*r* = 0.461, *p* < 0.001), while negative correlation was found with PLT (*r* = -0.359, *p* < 0.001) (Fig. 1A). Additionally, in this group, the stages of fibrosis had positive correlations with age (*r* = 0.203, *p* < 0.001), ALT (*r* = 0.350, *p* < 0.001), AST (*r* = 0.452, *p* < 0.001) and GGT (*r* = 0.417, *p* < 0.001), while had negative correlations with PLT (*r* = -0.381, *p* < 0.001) (Fig. 1B).

In the HBeAg-negative group, ALT (*r* = 0.393, *p* = 0.012) and AST (*r* = 0.446, *p* < 0.001) were found to be positively correlated with the grades of liver inflammation, while negatively correlated with PLT (*r* = -0.365, *p* < 0.001) (Fig. 1C). In the same group, fibrosis stages had positive correlation with AST (*r* = 0.298, *p* < 0.001), while had negative correlation with PLT (*r* = -0.470, *p* < 0.001) (Fig. 1D). There was no correlation between the grades of liver inflammation (*p* = 0.865) or the stages of fibrosis (*p* = 0.096) and age in the HBeAg-negative group.

Among 256 patients who had quantified HBV DNA, no significant correlations were found between HBV DNA levels and the grades of liver inflammation and the stages of fibrosis, neither in HBeAg-positive nor HBeAg-negative patients.

### Factors associated with significant liver histological damage

As showing in Table 2, factors associated with significant liver inflammation and fibrosis were analyzed in patients. ALT ≥ 2 × ULN (*p* < 0.001, OR = 9.385) and PLT (*p* = 0.004, OR = 0.993) were independently associated with significant liver inflammation and ALT ≥ 2 × ULN (*p* < 0.001, OR = 5.749), PLT ( *p* = 0.001, OR = 0.993), age ≥ 30 years (*p* = 0.014, OR = 1.941) were independently correlated with significant liver fibrosis in HBeAg-positive patients. ALT ≥ 2 × ULN (inflammation: *p* < 0.001, OR = 4.328; fibrosis:* p* < 0.001, OR = 3.555) and PLT (inflammation: *p* = 0.007, OR = 0.992; fibrosis:* p* = 0.001, OR = 0.991) were independently associated with significant liver inflammation and fibrosis in the HBeAg-negative group.

**Table 2:** Multivariate analysis for predictors of significant liver inflammation and fibrosis

	**Clinical parameter**	**Significant inflammation**			**Significant fibrosis**		
		**OR**	**95%CI**	***p*-value**	**OR**	**95%CI**	***p*-value**
**HBeAg-positive**	Age						
	< 30 years	1.000			1.000		
	≥ 30 years	0.984	0.547–1.773	0.958	1.941	1.143–3.297	0.014
	Sex						
	Female	1.000			1.000		
	Male	1.044	0.533–2.045	0.900	0.896	0.497–1.617	0.716
	ALT						
	< 1 × ULN	1.000			1.000		
	1–2 × ULN	1.259	0.661–2.396	0.483	0.912	0.495–1.679	0.768
	≥ 2 × ULN	9.385	4.880–18.052	< 0.001	5.749	3.420–9.666	< 0.001
	PLT	0.993	0.988–0.998	0.004	0.993	0.988–0.997	0.001
**HBeAg-negative**	Age						
	< 30 years	1.000			1.000		
	≥ 30 years	0.820	0.418–1.608	0.563	0.999	0.539–1.851	0.996
	Sex						
	Female	1.000			1.000		
	Male	2.254	0.923–5.504	0.065	1.553	0.659–3.659	0.314
	ALT						
	< 1 × ULN	1.000			1.000		
	1–2 × ULN	1.798	0.681–4.752	0.237	1.740	0.700–4.326	0.234
	≥ 2 × ULN	4.328	2.071–9.042	< 0.001	3.555	1.847–6.844	< 0.001
	PLT	0.992	0.987–0.998	0.007	0.991	0.986–0.996	0.001

Binary logistic regression analysis with a forward approach was used for Multivariate analysis

The ULN for ALT was 40U/L

The serum HBV markers, including HBV DNA, Hepatitis B surface antigen (HBsAg), Hepatitis B e antigen (HBeAg), were not subjected to analysis, due to the lack of quantitative data. Between 1999 and 2003, most individuals only underwent qualitative testing of HBV markers

*ULN *Upper limit of normal*, PLT* Platelet, *ALT* Alanine transaminase, *CI* Confidence interval, *OR* Odds ratio

### Comparison of liver histopathology based on age and ALT levels

**Fig. 2:**
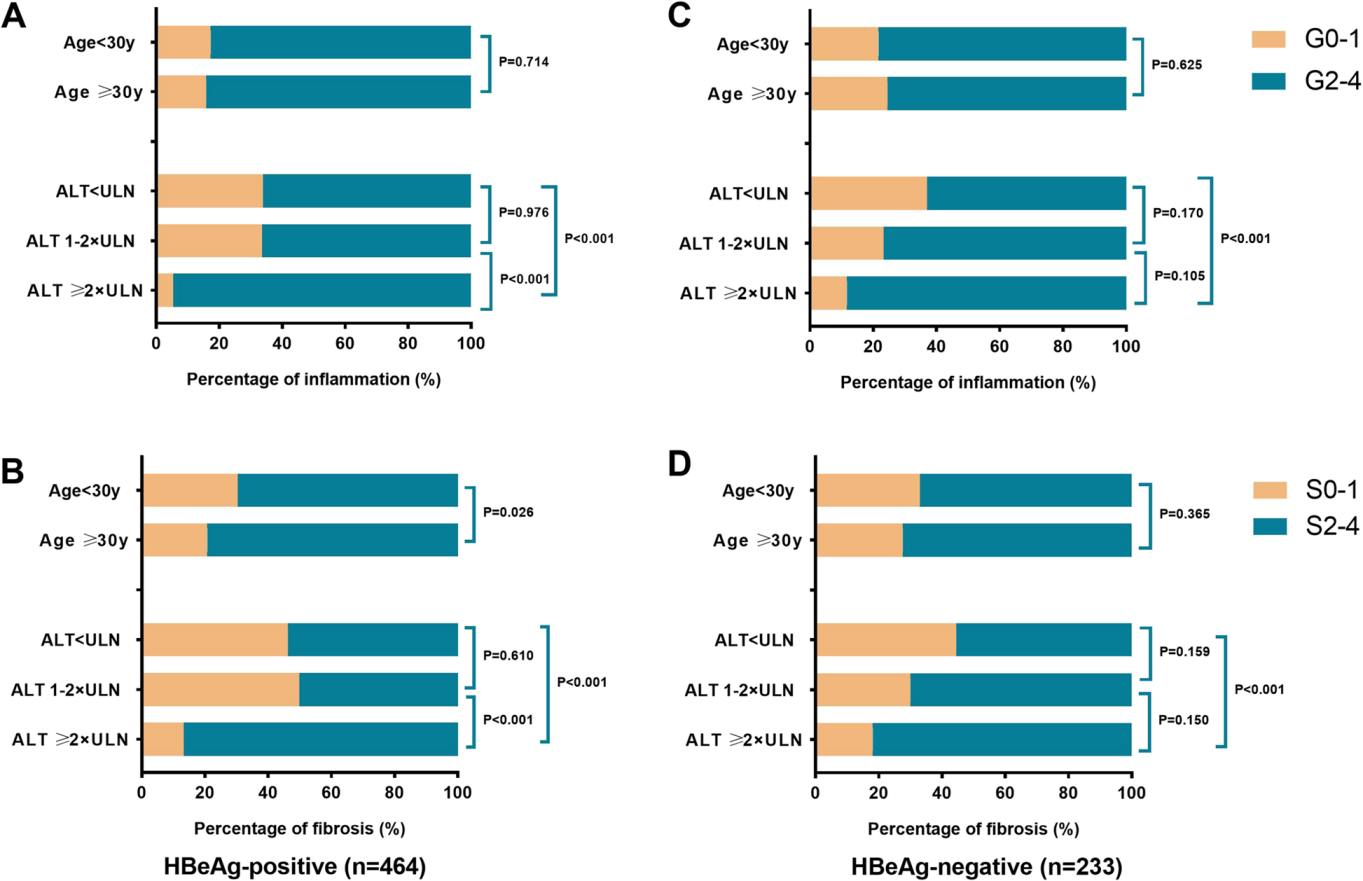
Distribution of liver inflammation and fibrosis based on age and the alanine transaminase (ALT) levels. **A** Distribution of liver inflammation based on age and ALT levels in patients with HBeAg-positive CHB. **B** Distribution of liver fibrosis based on age and ALT levels in in patients with HBeAg-positive CHB. **C** Distribution of liver inflammation based on age and ALT levels in in patients with HBeAg-negative CHB. **D** Distribution of liver fibrosis based on age and ALT levels in in patients with HBeAg-negative CHB. ALT, alanine transaminase; ULN, Upper limit of normal (40 U/L)

An age over 30 years and an elevated ALT level are the crucial determinants for initiating antiviral therapy. Figure 2 illustrates the incidence of significant liver inflammation and fibrosis among various age and ALT subgroups.

Among HBeAg-positive patients, the incidence of significant liver inflammation was detected in 82.8% and 84.1% of patients with aged < 30 years and aged ≥ 30 years (*p* = 0.714), and in 66.1%, 66.4% and 94.6% of patients with ALT < 1 × ULN, 1–2 × ULN, and ≥ 2 × ULN, respectively (Fig. 2A). Furthermore, significant liver fibrosis was detected in 69.6% and 79.2% of patients with aged < 30 years and aged ≥ 30 years (*p* = 0.026), and in 53.7%, 50.1% and 86.7% of patients with ALT < 1 × ULN, 1–2 × ULN, and ≥ 2 × ULN, respectively (Fig. 2B). Remarkable higher incidences of significant liver inflammation and fibrosis were observed in the subgroup with ALT ≥ 2 × ULN compared to the subgroup with ALT < 1 × ULN (inflammation: *p* < 0.001, fibrosis: *p* < 0.001) and subgroup with ALT 1–2 × ULN (inflammation: *p* < 0.001, fibrosis: *p* < 0.001). Notably, the incidences of significant liver inflammation (*p* = 0.976) and fibrosis (*p* = 0.610) in the subgroup with ALT < 1 × ULN were similar to those in the subgroup with ALT 1–2 × ULN.

Similarly, in HBeAg-negative patients, there is no significant differentiation in the incidence of significant liver inflammation (*p* = 0.625) and fibrosis (*p* = 0.365) between the subgroups with aged < 30 years and aged ≥ 30 years. However, the subgroups with ALT < 1 × ULN had significant lower incidence of significant liver inflammation and fibrosis than the subgroups with ALT ≥ 2 × ULN (inflammation: *p* < 0.001, fibrosis: *p* < 0.001), but was similar to ALT 1–2 × ULN. The incidence of significant liver inflammation was detected in 78.3% and 75.5% of patients with aged < 30 years and aged ≥ 30 years, and in 63.0%, 76.7% and 88.3% of patients with ALT < 1 × ULN, 1–2 × ULN, and ≥ 2 × ULN, respectively **(**Fig. 2C**)**. Furthermore, significant liver fibrosis was detected in 67.0% and 72.4% of patients with aged < 30 years and aged ≥ 30 years, and in 55.5%, 70.0% and 81.9% of patients with ALT < 1 × ULN, 1–2 × ULN, and ≥ 2 × ULN, respectively **(**Fig. 2D**)**.

Patients with ALT < 1 × ULN were also analyzed based on the normal clinical values for ALT ULN (i.e. 30 U/L for men and 19 U/L for women) proposed by Prati et al*.* There were 57 HBeAg-positive and 60 HBeAg-negative patients with ALT levels within the normal range, the distribution of liver histopathology is shown in Supplementary Fig. 2. Considering ALT ≤ revised ULN, significant liver inflammation and fibrosis were observed in 70.2% and 56.1% of HBeAg-positive patients and in 62.3% and 60% of HBeAg-negative patients, respectively. No significant difference was found in both inflammation and fibrosis between the two subgroups of patients.

### Comparison of liver histopathology in different age subgroups with persistently normal ALT levels

**Fig. 3:**
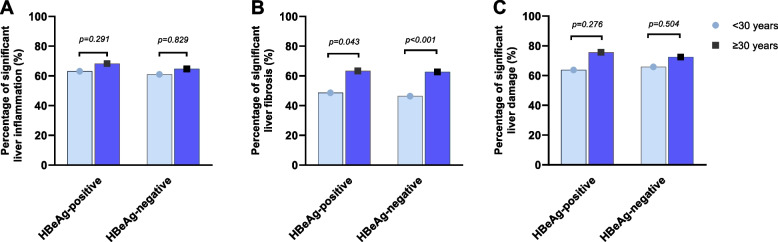
Incidence of significant liver inflammation and fibrosis in different ages of CHB patients with persistently normal ALT levels. **A** Incidence of significant liver inflammation in different ages of CHB patients with persistently normal ALT levels. **B** Incidence of significant liver fibrosis in different ages of CHB patients with persistently normal ALT levels. **C** Incidence of significant liver damage in different ages of CHB patients with persistently normal ALT levels. The liver damage was defined as either the grades of liver inflammation were exceeding G2 or the grades of liver fibrosis were exceeding S2

We depicted the overall distribution of liver histopathology in patients with persistently normal ALT levels (Supplementary Fig. 3). The incidence of significant liver inflammation and fibrosis were further analyzed in patients with persistently normal ALT levels according to different strata of ages (< 30 and ≥ 30 years), as shown in Fig. 3**.** In patients with ALT < 1 × ULN, the proportion of significant liver inflammation in patients aged < 30 years was comparable to that in patients aged ≥ 30 years, either in HBeAg-positive (≥ G2: 62.3% vs. 73.0%, *p* = 0.291), or in HBeAg-negative (≥ G2: 61.0% vs. 64.7%, *p* = 0.829) (Fig. 3A). Furthermore, the proportion of significant fibrosis of subgroups with aged < 30 years was significant lower in HBeAg-positive patients (≥ S2: 46.4% vs 67.6%, *p* = 0.043), and in HBeAg-negative patients (≥ S2: 46.3% vs 72.8%, *p* < 0.001), compared to those with aged ≥ 30 years (Fig. 3B). Broadly, in patients with persistently normal ALT levels, the percentage of those aged < 30 years and those aged ≥ 30 years who developed significant liver damage (either the grade of inflammation or the stage of fibrosis is above 2) is similar, either in HBeAg-positive (≥ G2 or ≥ S2: 63.8% vs. 75.8%, *p* = 0.276), or in HBeAg-negative (≥ G2 or ≥ S2: 65.9% vs. 72.5%, *p* = 0.504) (Fig. 3C).

## Discussion

In the present study, our investigation delved into the factors intertwined with liver histological grades among treatment-naïve patients. We analyzed the liver histology according to HBeAg status, ALT levels and age. We found that a negative correlation between liver histological grades and PLT, while ALT was positively correlated with it. Astonishingly, a substantial proportion, close to 70%, of CHB patients with persistently normal ALT levels, regardless of their age being above or below 30 years, experienced significant liver inflammation and fibrosis.

In this study, the majority of participants were male, which was consistent with previous studies [19, 20]. Previous studies have explored the causes of this phenomenon. A study indicated that the estrogen axis plays a protective role against HBV infection [21]. Other studies reported the direct binding of estrogen receptors and androgen receptors to binding sites within HBV enhancer I, the former inhibiting HBV transcription and the latter increasing overall HBV transcription [22, 23].

Liver biopsy is considered the definitive method for evaluating liver diseases, yet its routine implementation in clinical practice is limited due to its cost, invasiveness, and the potential risk of bleeding [24]. ALT is an enzyme found in hepatocytes, and an elevated ALT level often indicates hepatocellular damage [25]. There is no doubt that serum ALT level is a good indicator to gauge the severity of hepatitis. In lined with findings from the previous study, we identified a positive correlation between elevated serum ALT levels and both the grade of inflammation and stages of fibrosis [26, 27]. Notably, nearly all guidelines incorporate ALT level as a critical criterion for initiating antiviral therapy [13, 18], and it is recommended for CHB patients who had ALT exceeding 1 × ULN, especially those had ALT exceeding 2 × ULN. On the other hand, antiviral treatment is conservative for CHB patients with normal ALT, who were considered to be with no or minor liver damage. A retrospective study by Andreani et al. [28] showed only minimal liver damage in 40 patients with HBeAg-positive CHB and normal ALT levels. And Hui et al. [29] also revealed that patients with CHB and normal ALT levels suffered from less severe disease, and that the severity of that liver fibrosis did not significantly aggravate in those patients with normal ALT levels after 5 years of follow-up.

Nevertheless, a normal ALT does not always guarantee the absence of liver damage. A study demonstrated that among patients with HBeAg-positive CHB and normal ALT levels, 14.9% and 24.0% of participants exhibited significant liver inflammation and fibrosis, respectively, in contrast to 11.8% and 49.0% observed in those with HBeAg-negative CHB [19]. Another study conducted in Shanghai revealed that approximately 54% to 55% of CHB patients with relatively normal ALT levels presented with significant inflammation and fibrosis [30]. In the present study, our study identified a higher incidence of liver histological abnormalities in patients with persistently normal ALT levels. One of the reasons was that patients included in this study expressed a strong willingness for antiviral treatment, and most of them had a positive HBV DNA level. In fact, the detectable rate of HBV DNA in this study was probably underestimated according to the testing methods available at that time. On the other hand, before the neonatal hepatitis B vaccine program in China, HBV was predominantly transmitted vertically and histological damage in many CHB patients was initiated since early childhood. Another contributing factor could be the presence of CHB patients with normal ALT levels in an indeterminate phase, a commonly observed scenario.

Additionally, among these patients, we observed comparable proportions of significant liver inflammation and fibrosis between patients aged ≥ 30 years and those aged < 30 years, regardless of HBeAg status. In the current study, the incidence of liver histological damage was much higher than that report before. For instance, Cheng et al. [6] reported the incidence of significant liver inflammation was 22.6% and significant liver fibrosis was 9.8% in HBeAg-positive CHB patients aged ≤ 30. Tan et al. [31] demonstrated that among CHB patients who had age < 30 years and normal ALT levels, the prevalence of significant liver inflammation and fibrosis was 18.2% and 12.5% for HBeAg-positive patients, and 20.0% and 8.7% for HBeAg-negative patients, respectively. In summary, some CHB patients with persistently normal ALT levels and aged below 30 years may meet the criteria for antiviral therapy based on liver histology. It is worth considering whether antiviral therapy should be started earlier in such patients.

We also conducted an analysis to examine the correlation between liver histopathology and other biochemical parameters, including AST, GGT and PLT. Our finding revealed a positive correlation between AST and GGT with liver histopathology, while PLT showed a negatively correlation in HBeAg-positive patients. These results are aligned with previous study [32]. Furthermore, multivariate logistic regression analysis indicated that PLT was a risk factor for significant liver inflammation or fibrosis in CHB patients. The present study further supports the usefulness of several non-invasive methods such as aminotransferase-to-platelet ratio index (APRI), fibrosis 4 score (FIB-4) and gamma-glutamyl transpeptidase-to-platelet ratio (GPR), which have been employed in previous studies for evaluating liver advanced fibrosis or cirrhosis in CHB patients [33, 34]. The role of these non-invasive methods in assessing liver damage in CHB patients will be explored in future investigations.

This study had several limitations that should be acknowledged. Firstly, a limited number of patients had available and accurate HBV DNA and HBsAg results, which prevented us from conducting an analysis based on these specific parameters. Secondly, it is a single-center study, which may introduce selection bias and limit the generalizability of the findings. However, it is noteworthy that our sample size was relatively large. Currently, liver biopsy remains the gold standard for assessing liver diseases, however, there is a paucity of data on liver pathology with the utility of non-invasive methods. Thirdly, all patients in our study were of Asian ethnicity and their HBV genotypes were not investigated, because HBV genotyping is not routinely performed in clinical practice. However, it has been previously reported that predominant HBV genotypes in China are B and C. Lastly, the lack of follow-up after biopsy, hindered further evaluation of liver inflammation and fibrosis.

In conclusion, our findings indicate that a considerable proportion of patients with CHB and persistently normal ALT, including those below the age of 30 years, exhibited significant histological abnormalities. We recommend regularly assessing all CHB patients with normal ALT or aged < 30 years and starting antiviral therapy for them as early as possible.

### Supplementary Information

**Supplementary Material 1.**

**Supplementary Material 2.**

## Data Availability

The data analyzed for this study are available from the corresponding author, but restrictions apply to the availability of these data as they relate to the privacy of the patients and so are not publicly available. The data, however, will be available from the corresponding author upon a reasonable request.

## Abbreviations

CHB: Chronic hepatitis B
HBV: Hepatitis B virus
HCC: Hepatocellular carcinoma
NAs: Nucleoside (acid) analogues
ALT: Alanine transaminase
AST: Aspartate transaminase
GGT: Glutamyl transpeptidase
HBeAg: Hepatitis B e-antigen
HBsAg: Hepatitis B surface antigen
PLT: Platelet
ULN: Upper limit of normal
CI: Confidence interval
OR: Odds ratio
