# Supplementary material for: Patients with chronic hepatitis B who have persistently normal alanine aminotransferase or aged < 30 years may exhibit significant histologic damage
Source: BMC Gastroenterol. 2024 Mar 27;24:120. doi: 10.1186/s12876-024-03208-9 (PMC10967107; doi:10.1186/s12876-024-03208-9)

Supplementary Figure 1. Flow diagram of patient enrollment and reason for exclusion.

Supplementary Figure 2. The distribution of liver histology in patients with normal ALT was performed using the revised ULN (30 U/L for male ALT and 19 U/L for female ALT cutoff). ALT, alanine transaminase; ULN, Upper limit of normal.

Supplementary Figure 3. The distribution of liver histopathology in patients with persistently normal ALT.

Supplementary Figure 1

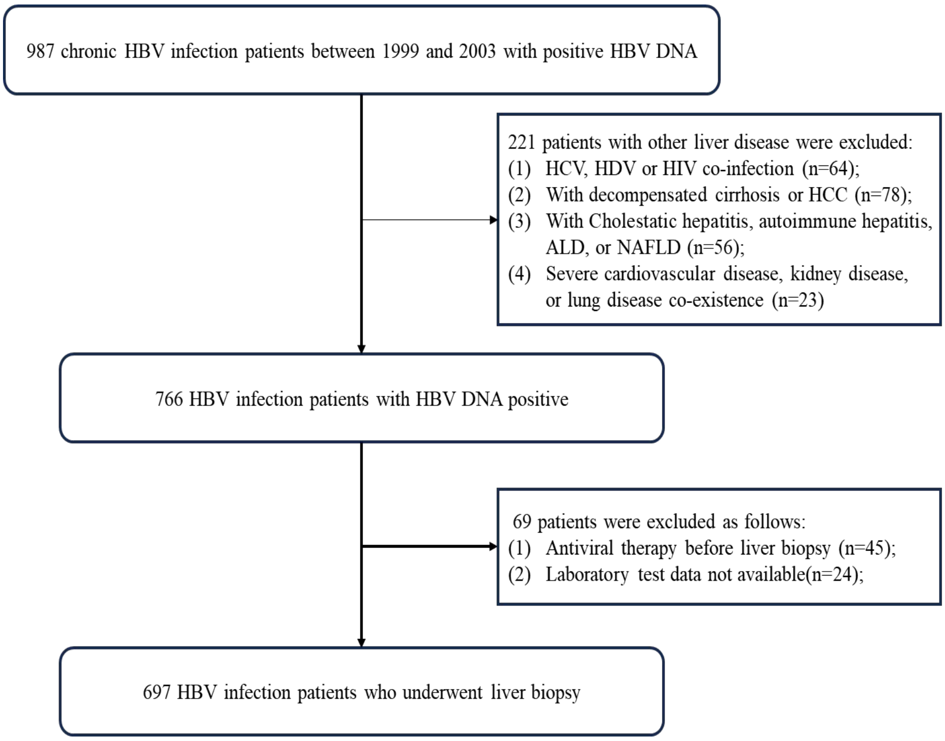

Supplementary Figure 2

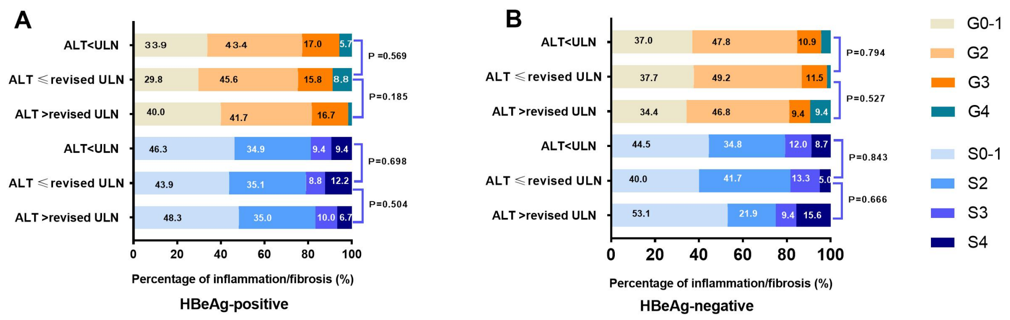

Supplementary Figure 3

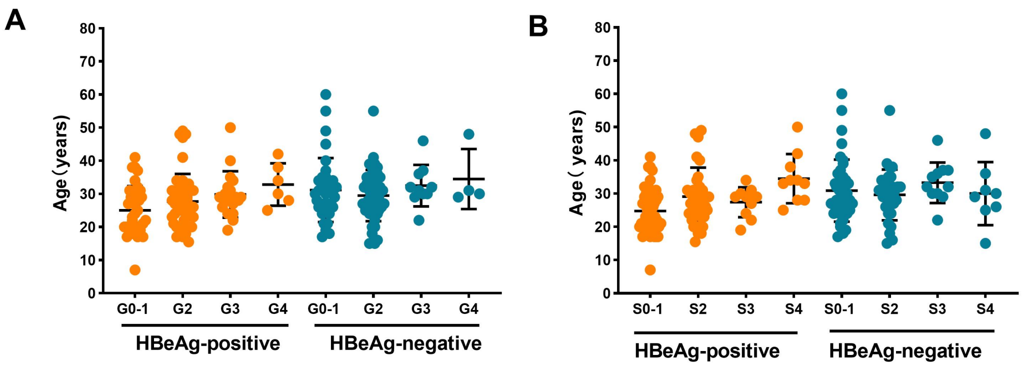

Supplement: Supplementary file 1 — Supplementary Material 1. [file 12876_2024_3208_MOESM1_ESM.pdf]
