# Supplementary material for: Patients with chronic hepatitis B who have persistently normal alanine aminotransferase or aged < 30 years may exhibit significant histologic damage
Source: BMC Gastroenterol. 2024 Mar 27;24:120. doi: 10.1186/s12876-024-03208-9 (PMC10967107; doi:10.1186/s12876-024-03208-9)
Supplement: Supplementary file 2 — Supplementary Material 2. [file 12876_2024_3208_MOESM2_ESM.docx]

| Characteristics | Total  (n=256) | HBeAg-positive | |  | HBeAg-negative | |
| --- | --- | --- | --- | --- | --- | --- |
|  |  | **Chronic infection (n=16)** | **Chronic hepatitis**  **(n=78)** |  | **Chronic infection**  **(n=2)** | **Chronic hepatitis (n=37)** |
| Inflammation n (%) |  |  |  |  |  |  |
| G0-1 | 46 (18.0) | 4 (25.0) | 5 (6.4) |  | 1 (50.0) | 5 (13.5) |
| G2 | 127 (49.6) | 10 (62.5) | 43 (55.1) |  | 1 (50.0) | 18 (48.7) |
| G3 | 54 (21.1) | 2 (12.5) | 18 (23.1) |  | 0 (0.0) | 12 (32.4) |
| G4 | 29 (11.3) | 0 (0.0) | 12 (15.4) |  | 0 (0.0) | 2 (5.4) |
| Fibrosis n (%) |  |  |  |  |  |  |
| S0-1 | 63 (24.６) | 5 (31.3) | 11 (14.1) |  | 1 (50.0) | 8 (21.6) |
| S2 | 117 (45.7) | 8 (50.0) | 44 (56.5) |  | 0 | 14 (37.9) |
| S3 | 36 (14.1) | 1 (12.5) | 9 (11.5) |  | 0 | 9 (24.3) |
| S4 | 40 (15.6) | 2 (6.2) | 14 (17.9) |  | 1 (50.0) | 6 (16.2) |

**Supplementary Table 1.** Distribution of liver inflammation grades and fibrosis stages in the four stages.
